# Expression quantitative trait loci for *ETV4* and *MEOX1* are associated with adult asthma in Japanese populations

**DOI:** 10.1038/s41598-021-98348-3

**Published:** 2021-09-22

**Authors:** Yohei Yatagai, Hisayuki Oshima, Tohru Sakamoto, Rie Shigemasa, Haruna Kitazawa, Kentaro Hyodo, Hironori Masuko, Hiroaki Iijima, Takashi Naito, Takefumi Saito, Tomomitsu Hirota, Mayumi Tamari, Nobuyuki Hizawa

**Affiliations:** 1 Department of Pulmonary Medicine, Faculty of Medicine, University of Tsukuba, Ibaraki, Japan; 2 Tsukuba Medical Center, Ibaraki, Japan; 3 National Hospital Organization Ibaraki Higashi National Hospital, Ibaraki, Japan; 4 Research Center for Medical Science, The Jikei University School of Medicine, Tokyo, Japan

**Keywords:** Genetics, Immunology, Molecular biology, Medical research, Molecular medicine, Pathogenesis, Risk factors

## Abstract

ETS variant transcription factor 4 (ETV4) is a recently identified transcription factor that regulates gene expression-based biomarkers of asthma and IL6 production in an airway epithelial cell line. Given that *ETV4* has not yet been implicated in asthma genetics, we performed genetic association studies of adult asthma in the *ETV4* region using two independent Japanese cohorts (a total of 1532 controls and 783 cases). SNPs located between *ETV4* and *mesenchyme homeobox 1* (*MEOX1*) were significantly associated with adult asthma, including rs4792901 and rs2880540 (*P* = 5.63E−5 and 2.77E−5, respectively). The CC haplotype of these two SNPs was also significantly associated with adult asthma (*P* = 8.43E−7). Even when both SNPs were included in a logistic regression model, the association of either rs4792901 or rs2880540 remained significant (*P* = 0.013 or 0.007, respectively), suggesting that the two SNPs may have independent effects on the development of asthma. Both SNPs were expression quantitative trait loci, and the asthma risk alleles at both SNPs were correlated with increased levels of *ETV4* mRNA expression. In addition, the asthma risk allele at rs4792901 was associated with increased serum IL6 levels (*P* = 0.041) in 651 healthy adults. Our findings imply that ETV4 is involved in the pathogenesis of asthma, possibly through the heightened production of IL6.

## Introduction

Recently, by combining context-specific gene regulatory network analyses with gene expression data, ETS translocation variant 4 (ETV4) has been identified as a transcription factor that regulates gene regulatory networks highly relevant to disease processes in asthma^1^, including responses to corticosteroids, regulation of immune system processes, and innate immune responses. In addition, siRNA-based knock-down of *ETV4* in an airway epithelial cell line model has demonstrated a significant reduction of cytokine expression relevant to asthma, including IL6 and IL8.

ETV4 belongs to the PEA3 subfamily of ETS transcription factors. ETV4 and ETV5 have similar functions^2^ and play important roles in a wide range of cellular processes^3–5^. It has been reported that IL6/STAT3-induced ETV5 promotes Th17 differentiation^6^. Th17 cells express high levels of MEK1^7^, which also induces ETV4 and ETV5^4^. Enhanced IL17 secretion from Th17 cells promotes IL6 production in bronchial epithelial cells^8^. These mechanisms may form a positive feedback loop, leading to the development of asthma via IL6 and Th17 signaling pathways including ETV4.

As *ETV4* has not been reported in any genetic studies of asthma to date, we performed a candidate gene association study of the *ETV4* gene with asthma in Japanese patients with adult asthma. We identified six SNPs significantly associated with asthma, including rs4792901 and rs2880540. These two SNPs were correlated with mRNA expression levels of *ETV4* and *Mesenchyme Homeobox 1* (*MEOX1*); asthma risk alleles at the two SNPs showed increased levels of mRNA expression of these genes. We further demonstrated that asthma risk allele (C) of rs4792901 was associated with increased levels of serum IL6 in non-asthmatic healthy adults.

## Results

The characteristics of two independent populations, Tsukuba cohorts 1 and 2, are shown in Table 1. Compared with non-asthmatic healthy adults, lower pulmonary function and a higher prevalence of atopy were consistently found in these asthma cases. Smoking status (never, ex, or current) was also significantly different between the cases and the controls in both cohorts.

**Table 1:** Characteristics of the study cohorts.

	Tsukuba Cohort 1		Tsukuba Cohort 2	
	Healthy controls	Asthma cases	Healthy controls	Asthma cases
Number of participants, n	967	242	565	541
Female sex, n (%)	526 (54.4)	143 (59.1)	271 (48.0)	302 (55.8)
Age, y (range)	50.0 (27–74)	51.2 (20–75)	53.5 (22–78)	61.4 (19–90)
Age of asthma onset, y (range)		37.5 (0–70)		42.4 (1–87)
**Smoking status, n (%)**				
Never-smoker	607 (62.8)	196 (81.3)	252 (44.6)	278 (52.9)
Ex-smoker	199 (20.6)	14 (5.8)	202 (35.8)	205 (39.0)
Current smoker	161 (16.6)	31 (12.9)	111 (19.6)	43 (8.2)
**Pulmonary function**				
FEV_1_ predicted, mean ± SD%	93.3 ± 12.1	89.9 ± 20.0	90.0 ± 13.7	81.6 ± 24.1
FEV_1_/FVC, mean ± SD%	83.2 ± 5.2	74.9 ± 11.1	81.3 ± 6.6	69.2 ± 13.2
Atopy (%)	55.9	73.0	62.1	68.7

In asthma cases in Tsukuba cohort 1, information on smoking status, pulmonary function, and atopy was missing in 1, 5, and 27 individuals, respectively. In healthy controls in Tsukuba cohort 2, information on pulmonary function was missing in 5 individuals. In asthma cases in Tsukuba cohort 2, information on age of asthma onset, smoking status, pulmonary function, and atopy was missing in 39, 15, 43, and 91 individuals.

**Figure 1:**
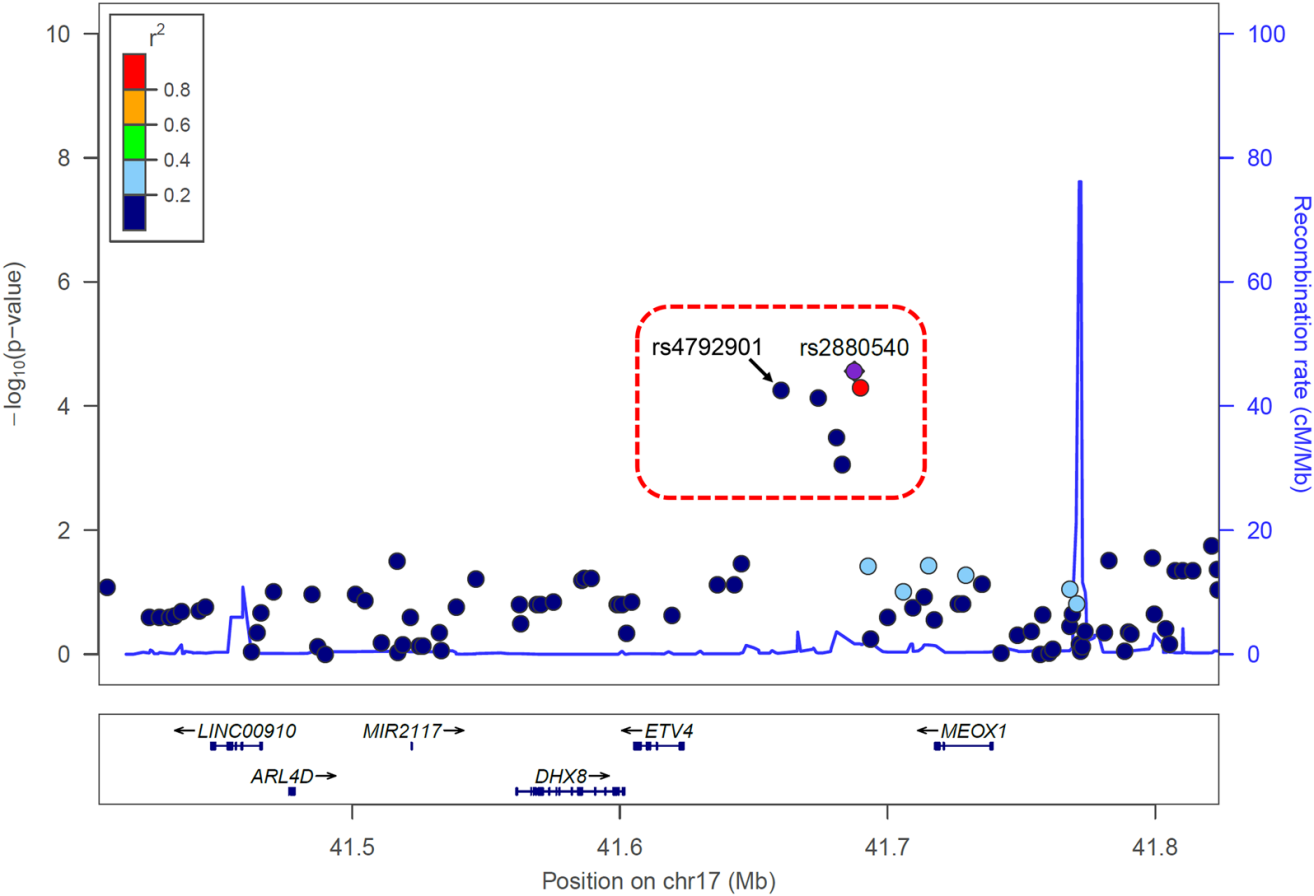
Association of SNPs in the *ETV4* region with asthma. The association study was carried out by logistic regression analysis adjusted for gender, age, and smoking status (never, ex, or current). The associations of the SNPs in a region spanning 200 kb upstream and downstream of the *ETV4* gene are plotted. The position on chromosome 17 is shown on the x-axis. The y-axis shows the statistical significances (− log_10_[*P*-value]). The purple diamond is the most significant rs2880540 SNP. All the other circle SNPs are color-coded according to the levels of linkage disequilibrium (LD) with rs2880540. The Genome Build/LD population is hg19/1000 Genomes Nov 2014 ASN. The relative location of genes and the direction of transcription are shown in the lower portion. The red dotted-line square indicates a cluster of six SNPs significantly associated with asthma.

Individuals in both cohorts had genome-wide genotyping. Because the genotyping platforms used were different between the cohorts, genotype imputations in a region spanning 200 kb upstream and downstream of the *ETV4* gene were performed for both cohorts based on each other’s genotypes as the reference. Figure 1 shows regional visualization of the meta-analysis of case–control association studies of Tsukuba cohorts 1 and 2. Ninety-nine SNPs were genotyped or imputed in the *ETV4* region in both cohorts. There was a cluster of six SNPs significantly associated with asthma between the *ETV4* and *MEOX1* genes. Table 2 shows the results of association studies of these six SNPs. Even after Bonferroni’s correction, five SNPs, not including for rs12603963, were significantly associated with asthma in the meta-analysis (*P* < 5.0E−4). Table S1 indicates case–control association studies and meta-analyses of all 99 SNPs.

**Table 2:** Case–control association studies and meta-analysis.

SNP	Major/Minor allele	Tsukuba cohort 1		Tsukuba cohort 2		Meta-analysis	
		OR* (95% CI*)	*P*	OR* (95% CI*)	*P*	OR* (95% CI*)	*P*
rs4792901	C/A	0.742 (0.598–0.921)	0.00679	0.751 (0.627–0.899)	0.00181	0.755 (0.659–0.866)	5.63E-5
rs1320304	T/C	0.746 (0.601–0.926)	0.00793	0.759 (0.634–0.909)	0.00270	0.759 (0.662–0.870)	7.47E-5
rs1613373	T/C	0.787 (0.636–0.973)	0.0270	0.765 (0.640–0.915)	0.00342	0.780 (0.681–0.893)	3.22E-4
rs12603963	T/C	0.857 (0.688–1.067)	0.1673	0.740 (0.617–0.888)	0.00119	0.790 (0.687–0.907)	8.67E-4
rs2880540	T/C	1.365 (1.097–1.699)	0.00521	1.358 (1.112–1.657)	0.00264	1.368 (1.182–1.584)	2.77E-5
rs4793006	C/T	1.345 (1.081–1.673)	0.00785	1.348 (1.104–1.645)	0.00336	1.354 (1.169–1.568)	5.04E-5

Associations between each SNP and asthma were examined by logistic regression analysis adjusted for gender, age, and smoking status (never, ex-, and current smoker).

*OR* odds ratio, *CI* confidence interval.

*ORs and 95% CIs were calculated by using a major allele as the reference.

**Figure 2:**
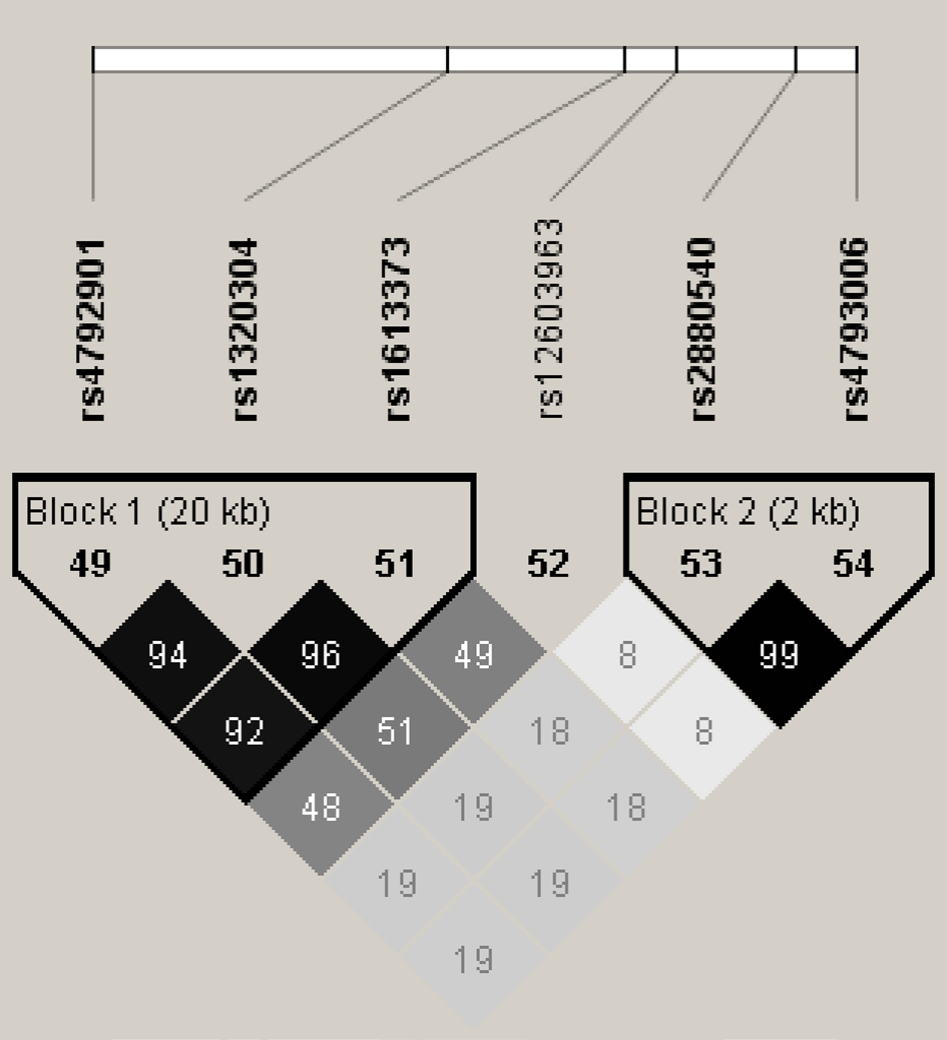
Linkage disequilibrium (LD) map and haplotype block structure. The pairwise LD map and haplotype blocks were constituted using the cluster of six SNPs. The SNPs are in the same order as the genomic organization on chromosome 17. Values of r^2^ are indicated in each cell. The regions surrounded by a black line are haplotype blocks.

Linkage disequilibrium (LD) and haplotype blocks in the six SNPs were estimated using all participants in both cohorts (Fig. 2). The six SNPs were divided into two haplotype blocks (Blocks 1 and 2). Rs4792901, rs1320304, and rs1613373 belonged to one haplotype block (Block 1). Rs2880540 and rs4793006 belonged to the other haplotype block (Block 2). Rs12603963 existed independent of the other five SNPs. In the meta-analysis of association studies (Table 2), rs4792901 (OR = 0.755, *P* = 5.63E−5) and rs2880540 (OR = 1.368, *P* = 2.77E−5) were the most significant in Blocks 1 and 2, respectively. The degree of LD between these two SNPs was r^2^ = 0.19. Table S2 and S3 demonstrate logistic regression analyses for each of these SNPs and cohort separately using the other SNP as a covariate. In these separate analyses, the meta-analysis of association studies of both rs4792901 and rs2880540 remained significant (*P* = 0.013 and 0.007, respectively) and identical effects were separately found in Tsukuba cohorts 1 and 2, suggesting that rs4792901 and rs2880540 may have independent effects on the development of asthma.

The major allele C of rs4792901 and the minor allele C of rs2880540 were found to be risk alleles for the development of asthma. The frequencies of haplotypes consisting of these two SNPs were estimated, and the association of the haplotypes with asthma was evaluated (Table 3); the CC haplotype consisting of rs4792901 and rs2880540 was strongly associated with asthma (OR = 1.399, *P* = 8.43E−7).

**Table 3:** Distribution of haplotypes consisting of rs4792901 and rs2880540 between the cases and controls.

Haplotype rs4792901-rs2880540	Total frequency	Case group ratio n (%)	Control group ratio n (%)	χ^2^	OR	*P*
AT	0.379	540.6 (34.5)	1211.9 (39.6)	11.151	0.796	8.00E−4
CT	0.327	500.4 (32.0)	1013.1 (33.1)	0.581	0.951	0.446
CC	0.276	502.6 (32.1)	773.9 (25.3)	24.258	1.399	8.43E−7
AC	0.019	22.4 (1.4)	65.1 (2.1)	2.695	0.668	0.101

*OR* odds ratio.

**Figure 3:**
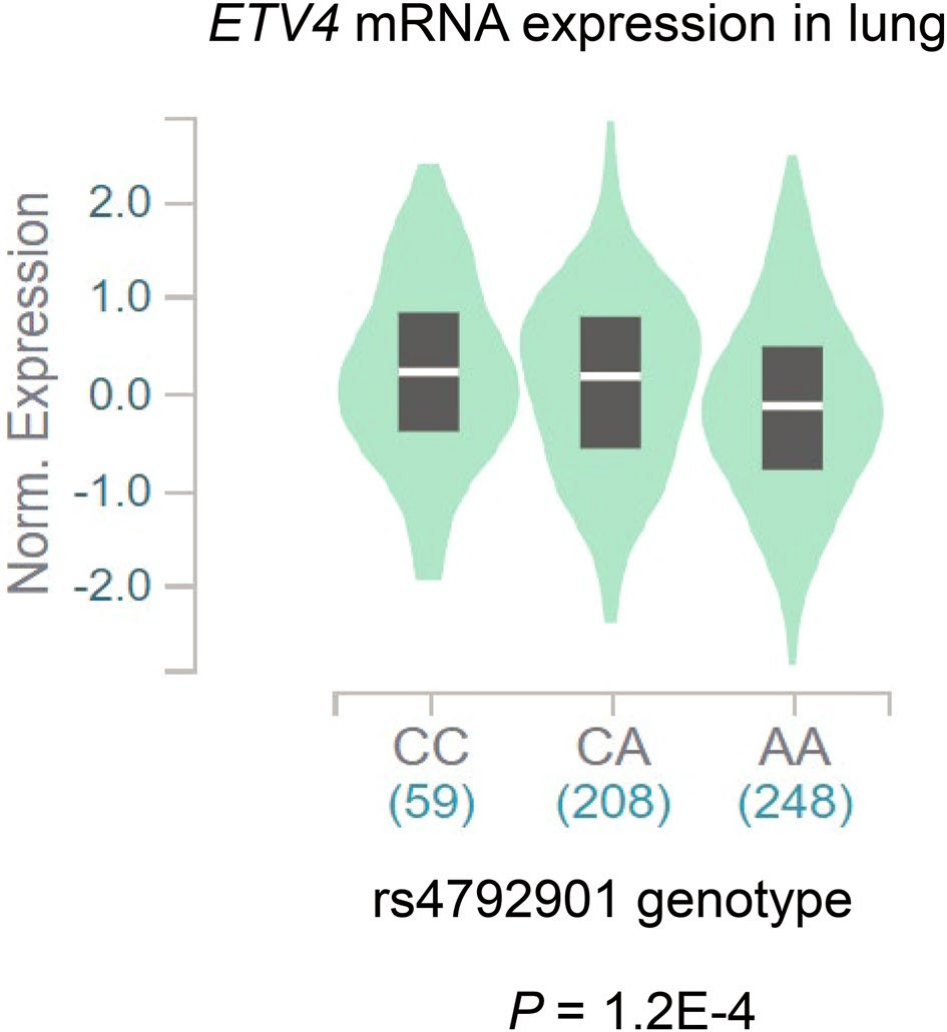
Expression quantitative trait loci (eQTL) analysis. The Genotype-Tissue Expression (GTEx) dataset includes 54 different non-diseased human tissue sites. The rs4792901 genotype is significantly associated with *ETV4* mRNA expression in lungs. The width of the violin plots indicates the sample density. The black boxes in the center represent the interquartile range. The white bars in the middle are the median values.

We performed *cis*-expression quantitative trait loci (eQTL) analysis in the Genotype-Tissue Expression (GTEx) project^9^ to verify the relationship between these two SNPs and mRNA expression levels. Rs4792901 was found to be associated with *ETV4* mRNA expression in tissues of lung, artery, skin, cultured fibroblasts, adipose tissue, prostate, and nerve. Rs2880540 was found to be associated with *ETV4* mRNA levels in adipose tissue. The C alleles of rs4792901 and rs2880540, both of which are asthma risk alleles, were significantly associated with higher levels of *ETV4* mRNA in lungs (*P* = 1.2E−4) (Fig. 3) and adipose tissue (*P* = 6.2E−7), respectively. Rs2880540 was also found to be a *cis*-eQTL for *MEOX1* in skin, adipose tissue, and muscle. The C allele of rs2880540 was significantly associated with higher levels of *MEOX1* mRNA in skin (*P* = 3.6E−8).

**Figure 4:**
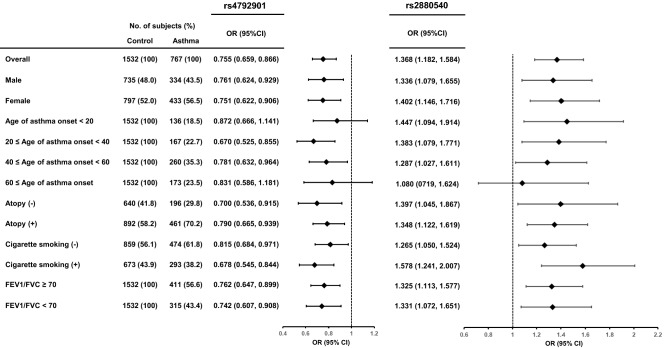
Stratified meta-analysis of association studies with asthma. Association of rs4792901 and rs2880540 with asthma was studied by logistic regression analysis based on an additive genetic model. The results were meta-analyzed using a random effects model. The width of the horizontal lines represents the 95 percent confidence intervals for each study.

Figure 4 shows a stratified meta-analysis of the association of these two SNPs with asthma according to gender, age at onset, the presence of atopy, smoking status, or the degree of airflow obstruction. None of these factors seemed to influence the genetic effect of the SNPs on the development of adult asthma.

**Figure 5:**
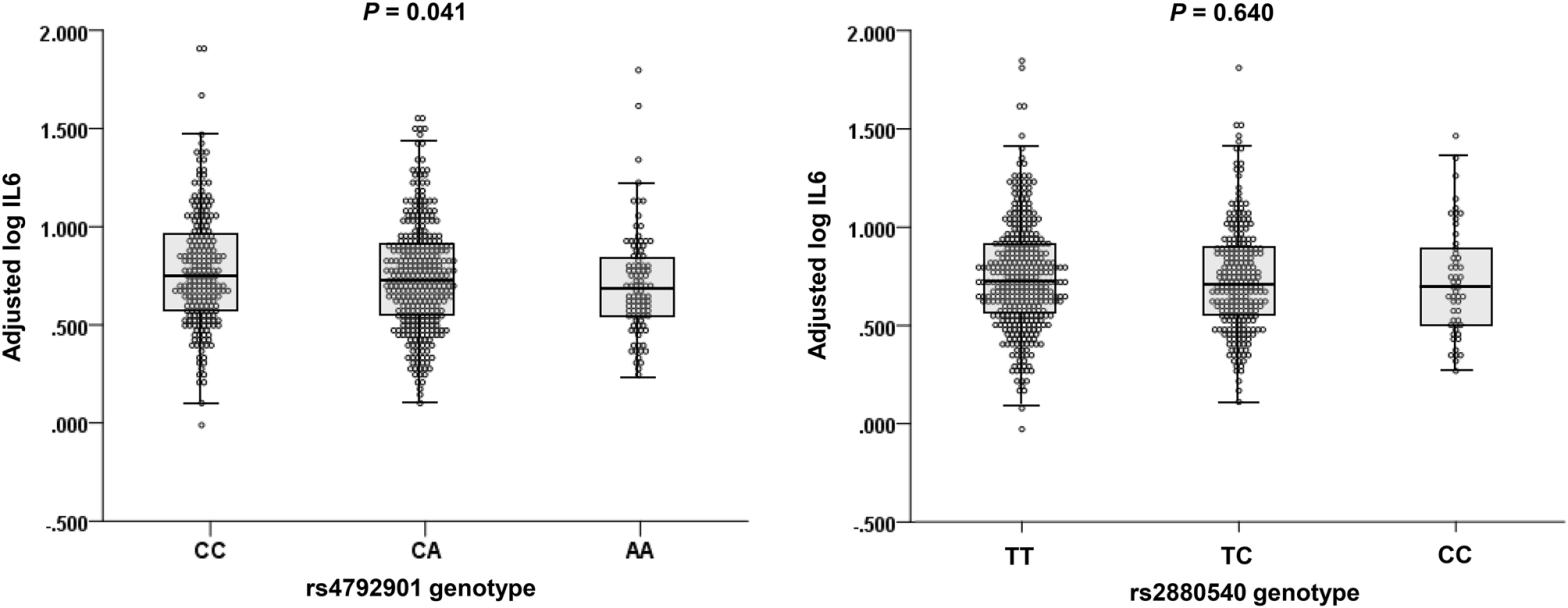
Scatter and box plots of adjusted serum log IL6 levels by rs4792901 and rs2880540. genotypes. The levels of IL6 were adjusted for gender, age, smoking status, and the other SNP genotype. The horizontal line in each box represents the median. The top and bottom box lines show the 25th and 75th percentiles, respectively. The whiskers show the maximum and minimum values, with the exceptions of outliers and extremes.

Serum IL6 concentrations (pg/ml) were determined in 651 healthy controls in Tsukuba cohort 1. Adjusting IL6 levels for gender, age, smoking status, and rs2880540 genotypes as independent variables revealed that rs4792901 was significantly associated with IL6 levels (unstandardized coefficient B = − 0.036, *P* = 0.041) (Fig. 5). The major allele C of rs4792901, a risk factor for the development of adult asthma, was associated with increased levels of serum IL6. No relationship was found between rs2880540 and IL6 levels (unstandardized coefficient B = − 0.009, *P* = 0.640). These results also suggested the possibility that the two SNPs independently influence the development of asthma.

## Discussion

In the present study, we focused on *ETV4* for a candidate gene association study of asthma, as ETV4 has been recently identified to be among the transcription factors that regulate multi-gene expression-based asthma biomarkers and increase the expression of IL6 and IL8 following stimulation with poly(I:C) in a nasal epithelial cell line model^1^. Although *ETV4* has not been detected in any genetic studies of asthma to date, in the present study, we successfully identified eQTLs for *ETV4/MEOX1* genes at 17q21.31 as significantly associated with adult asthma. Furthermore, we found that rs4792901 was associated with serum IL6 levels in non-asthmatic healthy adults. These findings support the hypothesis that *ETV4* is a novel asthma-susceptibility gene.

IL6 is involved in asthma pathogenesis through a variety of mechanisms^10^, including promotion of Th17 differentiation. In addition to *ETV4*, *MEOX1* (located in the vicinity of *ETV4*) is also a candidate molecule for the development of asthma via the Th17 pathway. Recently, it has been demonstrated that silencing of the CIP2A oncogene increases IL17 production in Th17 cells^11^. Transcription factors including MEOX1 and STAT3 are differentially expressed in CIP2A-silenced Th17 cells compared with control cells. Given that rs2880540, an eQTL for *MEOX1* as well as for *ETV4*, was associated with asthma independent of rs4792901, and the haplotype consisting of the two SNPs showed the strongest association with asthma, the combined effect of both genes may explain the findings in the current study.

Previous genome-wide association studies failed to identify the *ETV4*/*MEOX1* genes at 17q21.31 as a susceptibility region for asthma^12,13^; these studies primarily examined childhood-onset asthma, focusing on non-smokers only to exclude smoking-related airway diseases. In contrast, more than 80% of asthmatic patients in the current study were adult-onset; the mean ages of asthma onset in Tsukuba cohorts 1 and 2 were 37.5 and 42.4 years old, respectively. In fact, 70–80% of adult asthmatic patients are adult-onset in ethnic Japanese^14^, which is higher than in whites and blacks^15,16^. In addition, a substantial portion of participants in our study were smokers. Given that exposure to tobacco smoke leads to oxidative stress, increased mucosal inflammation, and increased expression of inflammatory cytokines such as IL6 and IL8^17^, the higher prevalence of patients with adult-onset asthma and smokers in this study may have allowed us to identify *ETV4* and *MEOX1* as candidate genes for asthma. In fact, even after excluding all cases with asthma onset age less than 18 years, the significant effects both of rs4792901 and rs2880540 on the development of adult asthma remained (Table S4).

ETV4 has been reported to be associated with multiple cancers^18^. MAPK and IL6/STAT3 signaling pathways play important roles in the development of many cancers^19,20^ as well as asthma^21,22^. To date, neither rs4792901 nor rs2880540 has been reported to be associated with cancer. In the future, the effect of these SNPs on the risk of cancer may need to be carefully investigated.

There are some limitations to this study. First, detailed information for type 2 inflammation including eosinophils in blood or sputum and fractional exhaled nitric oxide (FeNO) was not available in the present study. Since the IL6 and Th17 signaling pathways are mainly involved in type 2-low inflammation, which is more common in adult-onset asthma^23^, analyses with the more detailed information of type 2 inflammation might bring additional insights. Second, it might be possible that the imputation of genotypes is uncertain. However, imputation accuracy depends most strongly on genetic similarity between reference and target populations^24^, and all participants in both cohorts in this study are Japanese in the same geographical region, Ibaraki Prefecture. Third, in lungs, rs2880540 was not significantly associated with mRNA expressions of either *ETV4* or *MEOX1* in the GTEx project. Given the importance of systemic IL6 inflammation in severe asthma^25^, enhanced production of ETV4 in distant tissues, including adipose tissues, may be involved in the development of asthma. In any case, the functional consequences of this SNP in the lungs warrant study. Fourth, the results in this study were derived from a meta-analysis of two populations only. Replication of the results in other independent populations of adult asthma, including other ethnic groups, is necessary to support the generalizability of the findings in this study. Last, given the potential functional relevance of both *ETV4* and *MEOX1*, further studies are required to dissect the genetic contribution of this region and to determine whether a single causally associated variation can account for the genetic effect, or if multiple variants are independently involved.

In conclusion, we have demonstrated that two eQTLs in the *ETV4/MEOX1* region, rs4792901 and rs2880540, showed a significant association with the development of adult asthma in Japanese. These two SNPs are related to the expressions of *ETV4* and/or *MEOX1*, and these genes could be functionally related to the development of asthma. Further studies are merited to better understand the specific pathway mediating the role of these two genes in asthma pathogenesis.

## Methods

### Ethics statement

This study was approved by the Human Genome Analysis and Epidemiology Research Ethics Committee of the University of Tsukuba, the Tsukuba Medical Center, the Ibaraki Higashi National Hospital, and the Jikei University School of Medicine (Ethical approval number: H29-294). Written informed consent was obtained from each participant in accordance with the principles of the Declaration of Helsinki.

### Study participants

We studied two independent adult Japanese cohorts, Tsukuba cohorts 1 and 2. Tsukuba cohort 1 consisted of 967 healthy controls and 242 asthmatic patients. Tsukuba cohort 2 consisted of 565 healthy controls and 541 asthmatic patients. Healthy controls without respiratory diseases were recruited from persons who visited the Tsukuba Medical Center and the Health Center of Kamisu City for annual health checkups. Asthmatic patients were recruited from the University of Tsukuba Hospital and its affiliated hospitals^26^. Diagnoses of asthma were made based on the presence of recurrent episodes of two or more of the three symptoms (coughing, wheezing, and dyspnea) associated with demonstrable reversible airflow limitation and/or increased airway hyperresponsiveness to a bronchoconstrictor according to the criteria of the Japanese Society of Allergology^27^.

### Genotyping

We had genome-wide genotyping data determined by Illumina HumanHap 550v3/610-Quad BeadChips (Illumina, San Diego, CA, USA) for all the participants of Tsukuba cohort 1^28,29^. Whole-genome genotyping of Tsukuba cohort 2 was carried out by Infinium Asian Screening Array-24 v1.0 BeadChip (Illumina) involving 659,184 sequence variants using genomic DNA extracted from peripheral blood. Quality control for the SNPs was checked by PLINK 1.90 software^30^. None of the subjects were removed by a call rate for SNPs of < 0.1. SNPs with missing genotype rate > 0.1, minor allele frequency < 0.01, or Hardy–Weinberg equilibrium *P* value < 1.0 × 10^–6^ were excluded, leaving 448,612 SNPs.

Figure S1 shows principal component analysis (PCA) plots using all the genome-wide SNP data of Tsukuba cohorts 1 and 2, respectively. In both cohorts, PCA showed a single cluster, indicating there are no differences due to the structure of populations between cases and controls. Figure S2 shows quantile–quantile (Q-Q) plots showing the observed versus expected log *P*-values. The genomic inflation factors from the GWAS results of Tsukuba cohorts 1 and 2 were 1.0097 and 1.0334, respectively, indicating a low possibility of false-positive associations resulting from population stratification.

Because the genotyping platforms were different between Tsukuba cohorts 1 and 2, genotype imputation in the region spanning 200 kb upstream and downstream of *ETV4* of both cohorts was performed based on the other’s genotypes as the reference panel. We used a two-step imputation approach. First, imputation with pre-phasing of the target dataset was done. The haplotypes for each individual within the *ETV4* region were estimated using MACH software^31^ (pre-phasing). Then, the genotype imputation with pre-phased haplotypes in the MACH framework was done by Minimac3^32^. For post-imputation quality control, we selected SNPs with a Minimac r^2^ metric of ≥ 0.3.

### Expression quantitative trait (eQTL) analysis

We conducted a *cis*-eQTL analysis to examine the relationship between the identified SNPs and mRNA expression levels using the GTEx project version 8^9^ (https://gtexportal.org/home/). GTEx is the most comprehensive eQTL study. The recent GTEx dataset includes 54 different non-diseased human tissue sites and 17,382 RNA-Seq samples from 948 donors. In the GTEx dataset summary, *P*-values were calculated by a linear regression model between the genotypes and the expression levels.

### Determination of serum IL6 concentrations

Since it has been demonstrated that *ETV4* expression levels regulate IL6 production in a nasal epithelial line^1^, we analyzed the relationships between the two SNPs associated with *ETV4* expression and serum IL6 levels. Because the presence of asthma and asthma treatments may significantly influence the levels of serum IL6, we studied non-asthmatic healthy adults only. Serum samples of the healthy controls of Tsukuba cohort 1 were stored at − 80 °C. The IL6 concentrations (pg/ml) of the 651 samples were determined by chemiluminescent enzyme immunoassay at SRL, Inc. (Tokyo, Japan).

### Statistical analysis

In both Tsukuba cohorts 1 and 2, genotypes of the SNPs in a region spanning 200 kb upstream and downstream of the *ETV4* gene were examined for association with asthma by logistic regression analysis. The data were adjusted for gender, age, and smoking status (never, ex, or current). The results were then combined by meta-analysis using a random-effects model. These analyses were performed using IBM SPSS Statistics 26 (IBM Corp., Armonk, NY, USA). The local association results together with linkage disequilibrium coefficients and gene locations were visualized by LocusZoom^33^.

The LD block structure was examined by Haploview version 4.2^34^ using all the healthy controls and asthmatic patients in both cohorts. The r^2^ value for each pair of SNPs was calculated, and haplotype blocks were estimated. The pairwise haplotype frequencies were also estimated by Haploview. The frequencies of possible haplotypes were calculated using an implementation of expectation–maximization algorithm. For association studies, Haploview calculated simple χ^2^ for each haplotype.

Association of the SNP genotypes with serum IL6 levels was determined by multivariate linear regression analysis in IBM SPSS Statistics 26, assuming an additive genetic model adjusted for gender, age, smoking status (never, ex, or current), and the genotypes as independent variables. Since the serum IL6 concentrations followed a non-normal distribution, we performed a logarithmic transformation. In the additive genetic model, three genotypes including major allele homozygosity, heterozygosity, and minor allele homozygosity were separately evaluated. For scatter and box plots of serum IL6 levels among the SNP genotypes, the IL6 levels were adjusted using unstandardized coefficient B of each covariate in a multivariate linear regression analysis.

## Supplementary Information

Supplementary Information.

## Data Availability

Based on the “Act on the Protection of Personal Information” enforced in Japan and the conditions on which the informed consent was given, it is not permitted to disclose an individual’s genotypes and clinical information.
