# Supplementary material for: Expression quantitative trait loci for ETV4 and MEOX1 are associated with adult asthma in Japanese populations
Source: Sci Rep. 2021 Sep 22;11:18791. doi: 10.1038/s41598-021-98348-3 (PMC8458279; doi:10.1038/s41598-021-98348-3)
Supplement: Supplementary file 1 — Supplementary Information. [file 41598_2021_98348_MOESM1_ESM.pdf]

Expression quantitative trait loci for *ETV4* and *MEOX1* are associated with adult asthma in Japanese populations

Yohei Yatagai[1,*], Hisayuki Oshima[1,*], Tohru Sakamoto[1,*], Rie Shigemasa[1], Haruna Kitazawa[1], Kentaro Hyodo[1], Hironori Masuko[1], Hiroaki Iijima[2], Takashi Naito[2], Takefumi Saito[3], Tomomitsu Hirota[4], Mayumi Tamari[4] & Nobuyuki Hizawa[1]

[1]Department of Pulmonary Medicine, Faculty of Medicine, University of Tsukuba, Ibaraki, Japan. [2]Tsukuba Medical Center, Ibaraki, Japan. [3]National Hospital Organization Ibaraki Higashi National Hospital, Ibaraki, Japan. [4]Research Center for Medical Science, The Jikei University School of Medicine, Tokyo, Japan. *These authors contributed equally: Yohei Yatagai, Hisayuki Oshima and Tohru Sakamoto. *email: t-saka@md.tsukuba.ac.jp

Table S1. Case-control association studies and meta-analyses of all 99 SNPs.

SNP	Major/Minor allele	Tsukuba cohort 1			Tsukuba cohort 2			Meta-analysis	
		Genotyping (Typing rate)/ Imputation (Minimac r^2)	OR*	*P*	Genotyping (Typing rate)/ Imputation (Minimac r^2)	OR*	*P*	OR*	*P*
rs35612701	G/A	Imputation (0.411)	0.875	0.382	Genotyping (1.000)	0.854	0.151	0.858	0.0835
rs9646417	G/A	Genotyping (1.000)	1.077	0.502	Imputation (0.301)	1.134	0.315	1.098	0.255
rs11651341	T/C	Genotyping (1.000)	1.077	0.502	Imputation (0.302)	1.134	0.315	1.098	0.255
rs4534897	A/G	Genotyping (1.000)	1.077	0.502	Imputation (0.302)	1.134	0.315	1.098	0.255
rs4474733	G/T	Genotyping (1.000)	1.077	0.502	Imputation (0.302)	1.142	0.288	1.102	0.239
rs4793234	A/G	Genotyping (1.000)	1.077	0.502	Imputation (0.302)	1.161	0.230	1.110	0.203
rs11657004	A/G	Genotyping (1.000)	1.077	0.500	Imputation (0.305)	1.163	0.223	1.111	0.200
rs9912203	G/A	Genotyping (1.000)	1.080	0.484	Imputation (0.304)	1.175	0.190	1.117	0.174
rs4792990	A/G	Genotyping (1.000)	1.061	0.640	Imputation (0.710)	0.956	0.733	0.989	0.902
rs16940410	A/C	Genotyping (1.000)	0.906	0.515	Genotyping (1.000)	0.968	0.807	0.928	0.447
rs12600401	A/G	Genotyping (1.000)	1.206	0.0759	Imputation (0.467)	1.026	0.789	1.091	0.215
rs4793248	G/A	Genotyping (1.000)	0.789	0.0282	Imputation (0.451)	0.950	0.582	0.891	0.0976
rs147273156	T/C	Imputation (0.330)	0.391	0.204	Genotyping (0.999)	0.638	0.241	0.593	0.109
rs2086247	A/G	Genotyping (0.998)	1.042	0.731	Imputation (0.637)	0.929	0.550	0.973	0.750
rs75958943	C/A	Imputation (0.315)	1.266	0.434	Genotyping (1.000)	0.847	0.448	0.998	0.992
rs7215223	C/T	Genotyping (1.000)	1.192	0.0958	Imputation (0.536)	1.074	0.428	1.116	0.107
rs140343431	A/C	Imputation (0.342)	0.917	0.839	Genotyping (1.000)	0.586	0.0843	0.686	0.137
rs11079387	A/G	Imputation (0.529)	1.132	0.242	Genotyping (1.000)	0.975	0.780	1.032	0.645

rs7208829	G/A	Genotyping (1.000)	0.906	0.393	Imputation (0.470)	0.813	0.0526	0.845	0.0312
rs150322234	C/T	Imputation (0.326)	0.599	0.328	Genotyping (1.000)	1.188	0.537	0.978	0.922
rs4793250	G/A	Imputation (0.668)	0.810	0.130	Genotyping (1.000)	1.093	0.436	0.968	0.704
rs1984518	A/C	Genotyping (1.000)	1.122	0.294	Imputation (0.527)	1.076	0.452	1.085	0.255
rs9916834	C/T	Imputation (0.343)	1.275	0.278	Genotyping (0.998)	0.941	0.713	1.046	0.736
rs732508	A/G	Imputation (0.538)	1.133	0.254	Genotyping (0.999)	0.964	0.702	1.025	0.729
rs11868915	T/C	Imputation (0.716)	0.751	0.0500	Genotyping (0.998)	1.078	0.524	0.934	0.448
rs4632174	T/C	Imputation (0.355)	1.260	0.302	Genotyping (0.995)	0.919	0.597	1.023	0.862
rs12601607	A/G	Genotyping (1.000)	1.180	0.123	Imputation (0.833)	1.053	0.577	1.099	0.175
rs1463558	G/A	Genotyping (1.000)	0.852	0.200	Imputation (0.768)	0.863	0.153	0.863	0.0608
rs9898286	T/G	Genotyping (1.000)	1.199	0.0907	Imputation (0.868)	1.051	0.592	1.104	0.156
rs149226081	G/A	Imputation (0.506)	1.154	0.744	Genotyping (0.999)	0.579	0.0994	0.765	0.323
rs2127224	G/A	Genotyping (1.000)	1.199	0.0907	Imputation (0.873)	1.051	0.592	1.104	0.156
rs2279191	A/G	Genotyping (1.000)	1.199	0.0907	Imputation (0.877)	1.051	0.592	1.104	0.156
rs75388323	C/T	Imputation (0.333)	0.734	0.428	Genotyping (1.000)	0.784	0.232	0.771	0.143
rs2293014	A/G	Imputation (0.806)	0.876	0.267	Genotyping (1.000)	0.859	0.126	0.870	0.0639
rs8064459	G/A	Imputation (0.811)	0.876	0.267	Genotyping (1.000)	0.857	0.118	0.868	0.0602
rs13380802	G/A	Imputation (0.816)	0.876	0.267	Genotyping (0.999)	0.857	0.118	0.868	0.0602
rs2271957	T/C	Genotyping (1.000)	1.199	0.0907	Imputation (0.883)	1.051	0.592	1.104	0.156
rs2271958	G/A	Genotyping (1.000)	1.199	0.0907	Imputation (0.888)	1.051	0.592	1.104	0.156
rs9892162	T/C	Genotyping (1.000)	1.199	0.0907	Imputation (0.894)	1.051	0.592	1.104	0.156
rs1971	G/A	Genotyping (1.000)	0.947	0.628	Genotyping (0.999)	1.145	0.175	1.057	0.455

rs116868383	G/A	Imputation (0.484)	0.667	0.452	Genotyping (1.000)	0.605	0.168	0.644	0.142
rs145461557	C/T	Imputation (0.526)	1.154	0.744	Genotyping (1.000)	0.524	0.0598	0.720	0.237
rs12103666	G/A	Genotyping (1.000)	1.224	0.0560	Imputation (0.852)	1.078	0.421	1.132	0.0749
rs9899005	C/T	Genotyping (1.000)	1.224	0.0560	Imputation (0.853)	1.078	0.421	1.132	0.0749
rs17602795	C/T	Genotyping (1.000)	0.877	0.271	Imputation (0.879)	0.829	0.0581	0.852	0.0344
rs4792901	C/A	Genotyping (1.000)	0.742	0.00679	Imputation (0.909)	0.751	0.00181	0.755	5.63E-05
rs1320304	T/C	Genotyping (1.000)	0.746	0.00793	Genotyping (0.999)	0.759	0.00270	0.759	7.47E-05
rs1613373	T/C	Genotyping (1.000)	0.787	0.0270	Imputation (0.961)	0.765	0.00342	0.780	3.22E-04
rs12603963	T/C	Imputation (0.674)	0.857	0.167	Genotyping (0.998)	0.740	0.00119	0.790	8.67E-04
rs2880540	T/C	Genotyping (1.000)	1.365	0.00521	Imputation (0.764)	1.358	0.00264	1.368	2.77E-05
rs4793006	C/T	Genotyping (1.000)	1.345	0.00785	Imputation (0.762)	1.348	0.00336	1.354	5.04E-05
rs2074439	T/G	Genotyping (1.000)	0.786	0.0248	Imputation (0.894)	0.938	0.486	0.867	0.0381
rs565043	C/T	Genotyping (1.000)	0.950	0.642	Genotyping (1.000)	0.978	0.820	0.960	0.567
rs575873	T/C	Genotyping (1.000)	1.003	0.985	Imputation (0.616)	0.760	0.119	0.864	0.253
rs536652	C/T	Genotyping (1.000)	0.886	0.241	Imputation (0.855)	0.904	0.264	0.894	0.0976
rs556105	G/A	Genotyping (1.000)	0.899	0.537	Imputation (0.678)	0.807	0.194	0.851	0.177
rs479249	G/A	Genotyping (1.000)	0.841	0.340	Imputation (0.715)	0.819	0.232	0.825	0.117
rs8081000	A/G	Genotyping (1.000)	0.863	0.156	Imputation (0.878)	0.876	0.146	0.868	0.0370
rs55674920	G/A	Imputation (0.949)	0.902	0.363	Genotyping (0.999)	0.944	0.551	0.925	0.280
rs4793011	A/G	Genotyping (1.000)	0.900	0.346	Genotyping (0.998)	0.908	0.317	0.902	0.155
rs12944337	G/A	Imputation (0.992)	0.900	0.346	Genotyping (1.000)	0.908	0.317	0.902	0.155
rs3785806	C/T	Genotyping (1.000)	1.290	0.0540	Imputation (0.840)	1.137	0.315	1.195	0.0532

rs739769	G/A	Genotyping (1.000)	0.806	0.238	Imputation (0.818)	0.805	0.171	0.808	0.0728
rs739770	T/G	Genotyping (1.000)	0.806	0.238	Imputation (0.819)	0.805	0.171	0.808	0.0728
rs4793012	G/A	Genotyping (1.000)	1.061	0.563	Imputation (0.942)	0.972	0.748	1.004	0.956
rs1398882	C/T	Genotyping (1.000)	0.959	0.687	Imputation (0.953)	1.117	0.230	1.048	0.493
rs1405952	T/C	Genotyping (1.000)	0.974	0.804	Imputation (0.970)	0.936	0.492	0.945	0.430
rs9902563	C/T	Genotyping (1.000)	0.933	0.504	Imputation (0.985)	1.042	0.647	1.001	0.991
rs16940095	A/G	Genotyping (1.000)	0.891	0.559	Imputation (0.932)	0.802	0.223	0.852	0.231
rs1398883	A/G	Genotyping (1.000)	0.922	0.431	Genotyping (1.000)	1.040	0.659	0.995	0.943
rs11652705	G/A	Genotyping (1.000)	0.918	0.413	Imputation (0.952)	1.088	0.362	1.014	0.835
rs9901754	C/T	Genotyping (1.000)	0.921	0.418	Genotyping (1.000)	0.877	0.150	0.892	0.0891
rs9912855	A/C	Genotyping (1.000)	0.744	0.359	Imputation (0.845)	0.894	0.646	0.837	0.350
rs111627363	G/A	Imputation (0.968)	0.910	0.638	Genotyping (0.999)	0.789	0.197	0.847	0.224
rs72628310	C/T	Imputation (0.865)	1.259	0.0777	Genotyping (1.000)	1.039	0.764	1.139	0.154
rs12937489	C/T	Genotyping (0.999)	1.004	0.969	Genotyping (0.995)	0.943	0.551	0.971	0.683
rs9911324	G/T	Genotyping (1.000)	0.696	0.424	Imputation (0.670)	1.266	0.505	0.960	0.879
rs6503468	T/C	Genotyping (1.000)	1.091	0.392	Imputation (0.546)	0.927	0.414	0.978	0.747
rs1107748	T/C	Genotyping (1.000)	1.031	0.766	Imputation (0.566)	0.917	0.354	0.947	0.428
rs4793017	A/G	Genotyping (1.000)	1.049	0.644	Imputation (0.596)	0.908	0.303	0.949	0.446
rs8068071	A/G	Imputation (0.519)	0.905	0.335	Genotyping (1.000)	0.847	0.0772	0.861	0.0310
rs117538846	G/A	Imputation (0.582)	1.052	0.855	Genotyping (1.000)	0.990	0.970	1.030	0.878
rs7220711	A/G	Genotyping (1.000)	1.041	0.695	Imputation (0.600)	0.911	0.320	0.947	0.431
rs8082321	A/G	Imputation (0.429)	0.844	0.389	Genotyping (1.000)	0.965	0.796	0.923	0.472

rs4792909	G/T	Imputation (0.546)	0.901	0.323	Genotyping (1.000)	0.844	0.0769	0.857	0.0279
rs1877632	C/T	Genotyping (1.000)	0.919	0.472	Imputation (0.605)	0.937	0.526	0.912	0.226
rs17531431	C/T	Genotyping (1.000)	1.203	0.292	Imputation (0.465)	1.026	0.921	1.137	0.384
rs7216471	G/A	Genotyping (1.000)	1.065	0.756	Genotyping (1.000)	1.038	0.838	1.057	0.682
rs1513670	C/T	Genotyping (1.000)	1.086	0.431	Genotyping (1.000)	1.179	0.0662	1.145	0.0446
rs1534401	T/C	Imputation (0.993)	1.086	0.431	Genotyping (1.000)	1.179	0.0662	1.145	0.0446
rs9303537	G/T	Genotyping (0.999)	1.086	0.431	Imputation (0.992)	1.179	0.0662	1.145	0.0446
rs7222683	G/A	Genotyping (1.000)	0.860	0.157	Imputation (0.840)	0.851	0.0693	0.852	0.0178
rs9899889	G/T	Genotyping (1.000)	1.096	0.377	Imputation (0.962)	1.174	0.0736	1.146	0.0429
rs9910135	C/T	Imputation (0.826)	0.951	0.633	Genotyping (1.000)	0.858	0.0850	0.892	0.0901
rs865429	A/G	Genotyping (0.999)	1.024	0.847	Genotyping (1.000)	1.090	0.436	1.050	0.550
rs1237278	C/T	Imputation (0.758)	0.907	0.362	Genotyping (1.000)	0.903	0.282	0.899	0.129
rs1234612	T/C	Genotyping (1.000)	0.943	0.675	Genotyping (1.000)	1.077	0.561	0.994	0.946
rs4792912	C/T	Imputation (0.935)	0.881	0.403	Genotyping (1.000)	1.158	0.295	0.998	0.986
rs2074143	A/G	Imputation (0.930)	0.888	0.435	Genotyping (0.999)	1.158	0.295	1.003	0.980

Associations between each SNP and asthma were examined by logistic regression analysis adjusted for gender, age and smoking status (never, ex-, and current smoker). OR, odds ratio. *ORs were calculated by using a major allele as the reference.

Table S2. Logistic regression analysis adjusted by rs2880540 genotype, gender, age and smoking status

SNP	Tsukuba cohort 1		Tsukuba cohort 2		Meta-analysis	
	OR*	*P*	OR*	*P*	OR*	*P*
rs4792901	0.810	0.079	0.816	0.049	0.826	0.013

OR, odds ratio.

Table S3. Logistic regression analysis adjusted by rs4792901 genotype, gender, age and smoking status

SNP	Tsukuba cohort 1		Tsukuba cohort 2		Meta-analysis	
	OR*	*P*	OR*	*P*	OR*	*P*
rs2880540	1.294	0.068	1.225	0.075	1.251	0.007

OR, odds ratio.

Table S4. Meta-analysis of case-control association studies

SNP	Major/Minor allele	Meta-analysis (All individuals)		Meta-analysis (Age of asthma onset ≥ 18**)	
		OR* (95% CI*)	*P*	OR* (95% CI*)	*P*
rs4792901	C/A	0.755 (0.659-0.866)	5.63E-5	0.746 (0.640-0.869)	1.80E-4
rs2880540	T/C	1.368 (1.182-1.584)	2.77E-5	1.306 (1.107-1.546)	1.54E-3

Associations between each SNP and asthma were examined by logistic regression analysis adjusted for gender, age and smoking status (never, ex-, and current smoker). OR, odds ratio; CI, confidence interval.

*ORs and 95% CIs were calculated by using a major allele as the reference. **Number of cases with age of asthma onset ≥ 18 was 608.

Figure S1. Principal component analysis

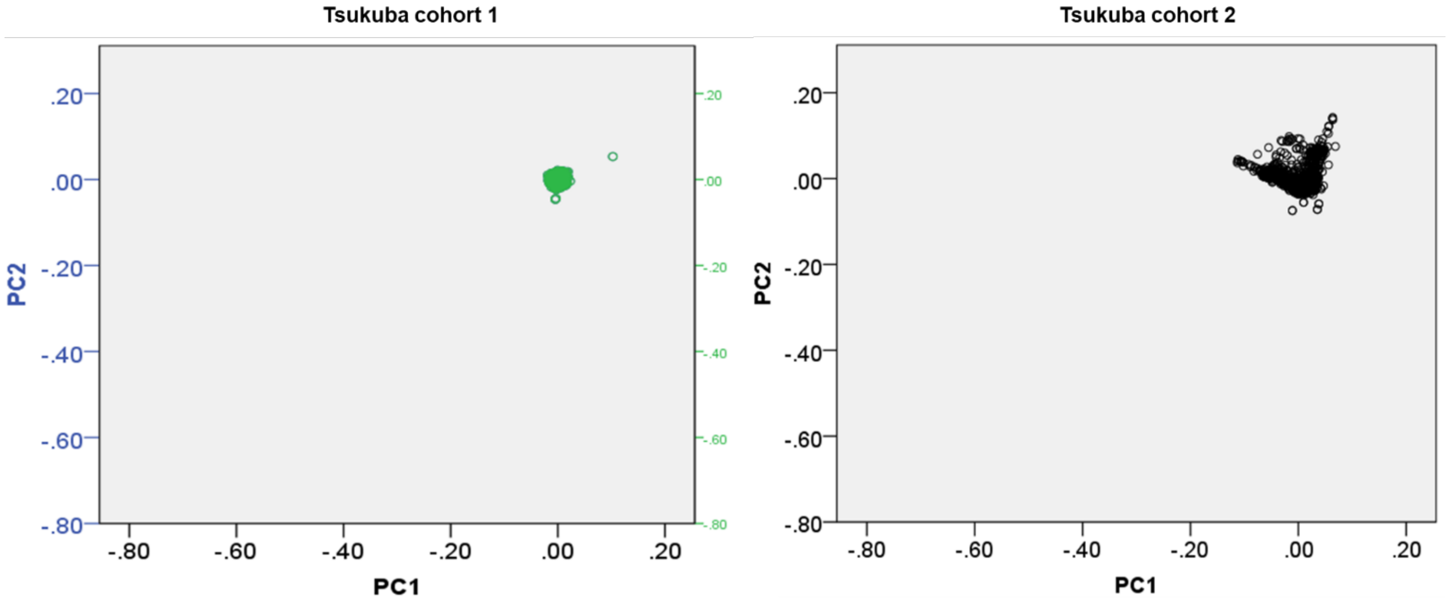

Each dot represents an individual participant. The x-axis represents the first principal component (PC1) and its percentage variance, and the y-axis represents the second principal component (PC2) and its percentage variance.

Figure S2. Quantile-quantile (Q-Q) plots of observed versus expected *P* values of the GWAS results

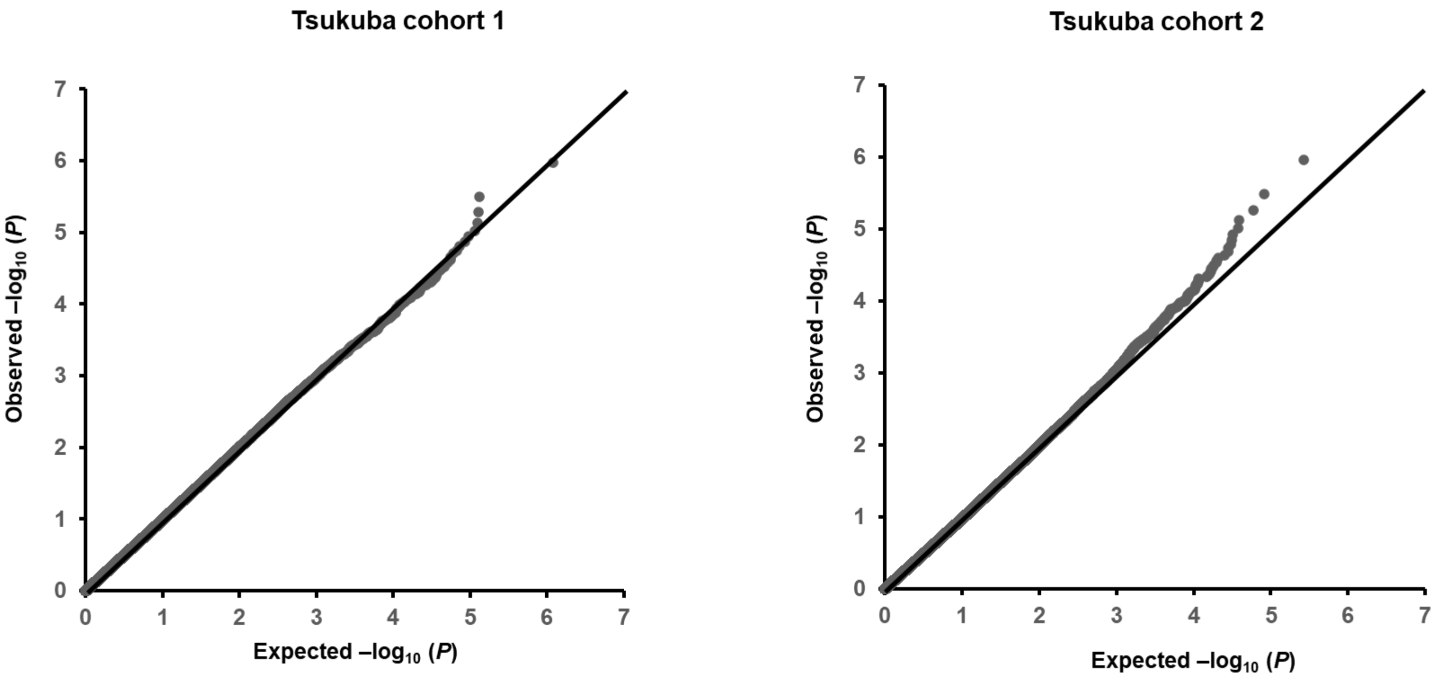

The straight line on the Q-Q plot indicates the distribution of SNP markers under the null hypothesis. The genomic inflation factors from the GWAS results of Tsukuba cohorts 1 and 2 are 1.0097 and 1.0334, respectively.
